# Role of neonatal team including nurses in prevention of ROP

**Published:** 2017-11-11

**Authors:** Srinivas Murki, Sandeep Kadam

**Affiliations:** 1 Chief Neonatologist: Fernandez Hospital, Hyderabad, India.; 2 Neonatologist: KEM Hospital, Pune, India.

**Neonatal nurses are pillars of the neonatal intensive care units (NICU). Their knowledge and clinical skills are essential in providing best practices in quality care in preventing ROP in preterm babies.**

**Figure:**
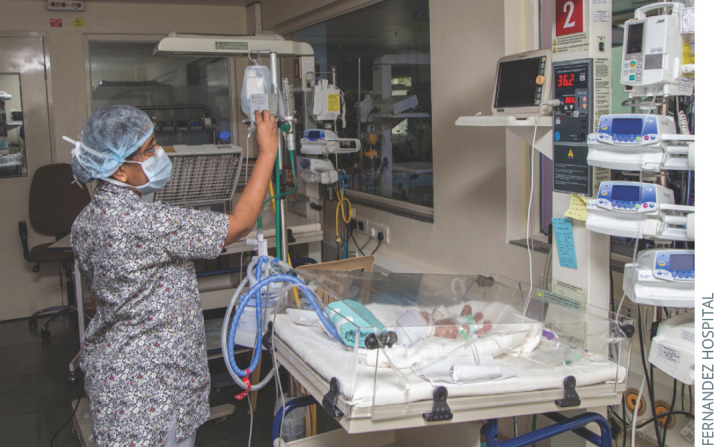
A nurse optimising oxygen for a preterm infant by adjusting the FiO2 with a blender at Fernandez Hospital. INDIA

Retinopathy of Prematurity (ROP) is a preventable cause of blindness in children. As smaller and sicker neonates are surviving in the neonatal intensive care unit (NICU), the incidence and severity of ROP is also on the rise. We are now facing the third epidemic of ROP; the first being in 1950s with liberal oxygen use, the second one in the developed world where smaller and smaller babies survived and the third epidemic in India/developing world, where ROP is additionally seen in bigger babies, for lack of optimal care and oxygen administration.^1^ It is time to identify the preventable causes of ROP and implement solutions that would result in reduction of incidence and severity of ROP. In this article we aim to identify the role of nurses and health staff in preventing ROP and in identifying at-risk babies in the NICU for effective screening of ROP.

## Prevention of ROP

Prevention of ROP includes improved care in the NICU. Improved care results in reduced morbidities and reduced risk factors that put a neonate at-risk for developing ROP. Improved neonatal care is the domain of pediatricians, medical officers, resident doctors and neonatal nurses who are involved in the care of these babies. Any intervention to improve quality care of a newborn can contribute to reducing the incidence of ROP in developing countries.

### Judicious oxygen therapy

Oxygen is a drug and it should be administered in a quantity that is appropriate to the need. Each neonatal care unit should have a written policy outlining appropriate use of oxygen therapy. Oxygen level in the blood should be continuously monitored using a pulsoximeter. A target of 90–95% SpO2 in all newborns on any respiratory support, including oxygen therapy, should be maintained.^2^ One should avoid 100% oxygen in the labour room and use a blender to target SpO2. The most important tool at hand today is control of oxygen saturation. It is also important to avoid fluctuations in SpO2 especially at high levels.

### Judicious use of blood transfusions

Transfusion of packed RBCs is another ROP risk factor. Adult haemoglobin has α2β2 chains which have low affinity for oxygen as compared to the haemoglobin of preterm babies, hence more tissue delivery of oxygen results in hyperoxia. Adult RBCs are rich in 2, 3 Diphosphoglyceric acid (DPG) and this binds with deoxyhemoglobin and stabilises the low oxygen carrier state making it difficult for oxygen to bind, resulting in more release of oxygen to the retinal tissue. Significantly low haemoglobin or platelets on the other hand can also worsen ROP. Hence written guidelines for transfusion in the NICU will help in restricting adult blood transfusions.

### Prenatal steroids

Use of prenatal steroids is a well-known approach to prevent respiratory distress and intraventricular hemorrhage, two important risk factors of ROP. All women expected to deliver between 24 to 34 weeks of gestation should be given a course of either betamethasone or dexamethasone intramuscularly at-least 24 hours before the delivery of the baby.^3^

### Nutrition

Postnatal weight gain predicts risk of retinopathy of prematurity. Poor weight gain in postnatal period increases the risk of severe ROP.^4^,^5^ Insulin-like growth factor 1 (IGF-1) controls VEGF-mediated vascular growth, which is important for retinal vasculature. Hence both increased nutrition and adequate IGF-1 concentrations seem to be necessary for postnatal growth and reduction in risk of ROP.^6^ Ensuring early administration of colostrum, exclusive and aggressive use of mothers own milk or donor milk, human milk fortification, kangaroo mother care, mothers involvement in baby care are some of the interventions in improving the nutritional status of preterm infant.

### Infections

Neonatal infections, particularly fungal infections, are also risk factors for ROP. A systematic review and meta-analysis of eight studies found that systemic fungal infection in very low birth weight infants was significantly associated with ROP and severe ROP.^7^ Neonatal bacteremia is associated with severe retinopathy of prematurity in extremely low gestational age neonates. The increased risk associated with infection might be partly due to systemic inflammation, which could act synergistically with hyperoxia. Chorioamnionitis is often associated with higher levels of circulating proinflammatory cytokines which could act with postnatal infections resulting in higher cytokines and later development of ROP. Some of the Do's and Don'ts in prevention of neonatal infections are described in Table 1.

To summarise, prevention of ROP by reducing risk factors that disrupt normal retinal vascularisation is likely to be more effective than late treatment of neovascularisation. This is not only with respect to vision, but also with other co-morbidities of a premature birth. Careful control of oxygen saturation, normalisation of serum IGF-1 concentrations, provision of adequate nutrition, curbing the negative effects of infection and inflammation, judicious use of oxygen in delivery room and the NICU, and a reduction in blood transfusion in the NICU could promote adequate postnatal growth and improve neural and vascular development of the retina. Nurses in the NICU are the backbone of all NICUs across the world. All the nurses should promote quality care and developmentally supportive care in the NICU.

### Secondary prevention of ROP: early case detection and treatment

The unit should have protocols that cover all steps of screening and management of ROP.

- The simplest method to ensure that all eligible infants are examined at an appropriate time is to identify them when they are first admitted to the NICU. A nurse can help by entering details into a book or electronic database, noting the date of the first examination and subsequent examinations. This helps in ensuring no baby is missed for ROP screening.
- Deciding when examinations are complete and organising timely treatment and long-term follow-up also remains a challenge. Nurses can help in establishing clear communication between neonatologists, resident doctors and ophthalmologist in the NICUs and, importantly, with parents.
- ROP prevention is a team responsibility, and parents must be seen as equal partners in that team. Good communication is at the heart of the relationship between the baby's present medical caregivers and the parents, the future caregivers. Nursing staff inevitably spend the maximum time talking to parents. They are often the most trusted members of the team, so their input into written material and how it is presented is vital.
- Good awareness of communication problems, clear-cut organisational responsibilities and most importantly, working closely with parents as equal partners should prevent most of the difficulties in ROP screening.

## Nurses role in prevention can be summarised in the following manner

### Specialist knowledge in clinical management

Nurses should be aware of all the risk factors known to be associated with ROP. They should form the core team in implementing good practices such as target oxygen saturation, encouraging breastfeeding, hand hygiene and asepsis to reduce infections, and support for nutrition to achieve good weight gain. These would help in reducing ROP in the unit.

### Clinical advocacy

Nurses are primary care givers in a neonatal ICU. They can also advocate for providing best practices in the NICU. Some of these include kangaroo care, thermal care, infection prevention and breastfeeding. Advocating for the adjustment of environmental factors (minimal handling, noise and light) and developmental care are core components of nursing. That will maximise the chances of healthier developmental outcomes in extremely preterm newborns, including vision, hearing and cognitive function.

### Leadership and mentoring

For sustainable change, leadership from within the nursing profession for policies on educational opportunities and competency-based training programmes is needed. Experienced nurses can coach the young nurses to improve quality care in the NICU.

**Figure:**
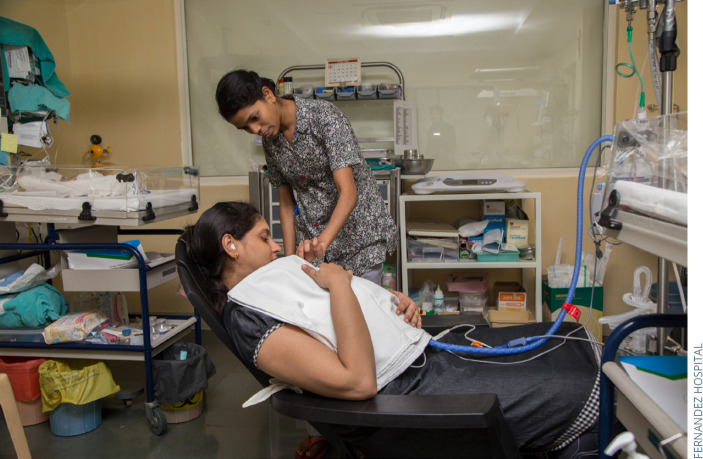
A nurse helping a new mother providing kangaroo mother care. INDIA

### Counseling

Nurses are key personnel who counsel parents regarding breast milk and kangaroo care. They provide emotional support and motivate parents in developmental care, adherence to ROP screening and follow-up. Using innovative strategies such as ROP appointment and follow up cards for parents or using mobile applications can ensure timely management of ROP.

**Figure:**
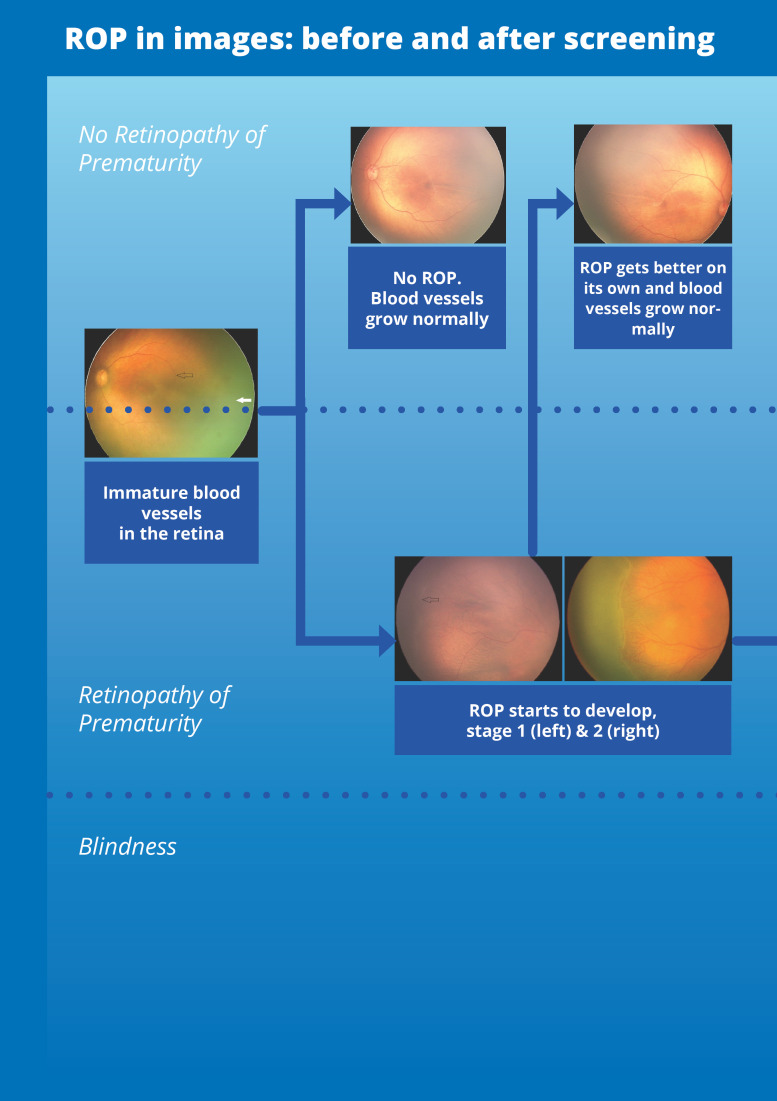


**Figure:**
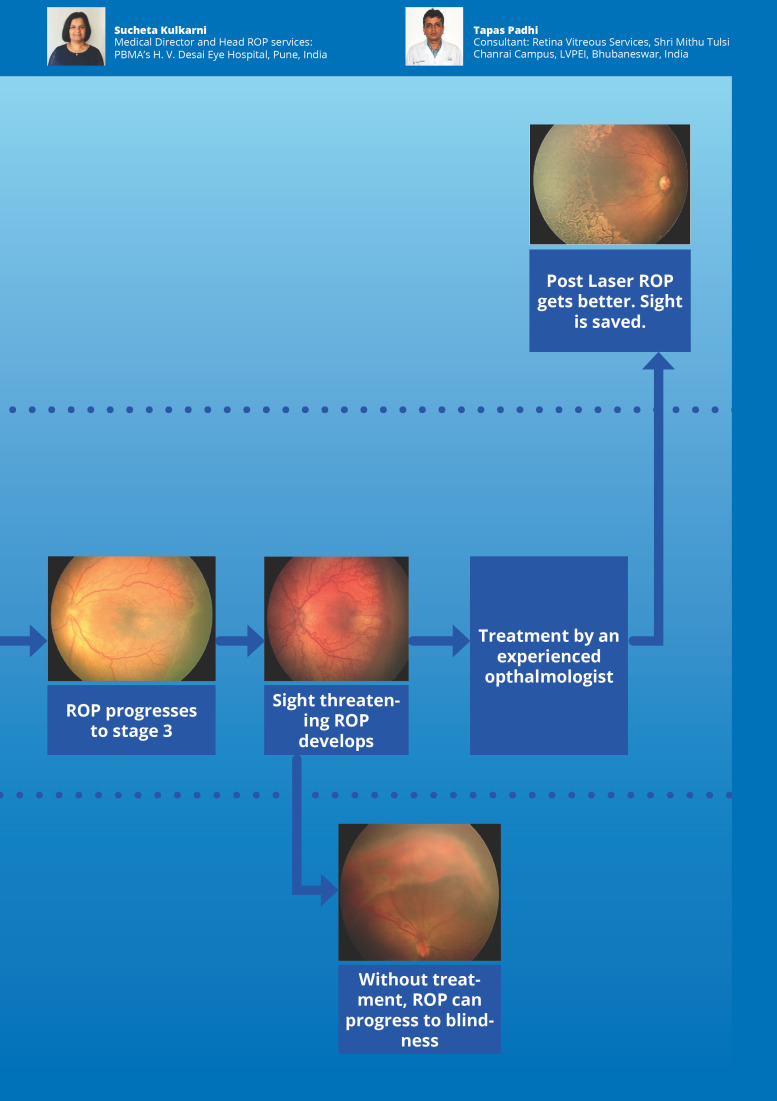


**Table 1:** Do's and Don'ts to prevent neonatal infections

Do's	Don'ts
Hand hygiene	Excessive use of antibiotics
Aggressive use of enteral feeds	Evasive ventilation
Restricted oxygen	Central lines
Bundles of care (eg. VAP, CLABSI)	
Optimal nurse: patient ratio	
Maternal participation	
Kangaroo care	
Good house keeping	

**Table 2:** Ready reckoner for nurses in prevention and management of ROP

Identify newborns at risk of ROP at admission to the NICU. Note the expected screening date and time on the case file. Encourage communication with the obstetrician for improving the coverage of antenatal steroid usage. Restrict oxygen use in the NICU. Monitor saturations in all babies on oxygen and set targets between 90 to 95%. Restrict usage and duration of antibiotics, intravenous fluids, parenteral nutrition and continuous positive airway pressure. Encourage mothers of preterm babies to use kangaroo mother care, continue with breastfeeding and aggressive enteral nutrition and developmentally supportive care. Co-ordinate with the neonatal and ophthalmology team in timely preparation of the newborn (pain relief and eye dilatation) for ROP screening. Monitor the newborn during the screening procedure. Play an active part in communication with the parents on screening outcomes and need for treatment when needed. At discharge brief the mother on the need for subsequent screening for ROP, hearing and neurodevelopment. Ensure follow up on schedule and become part of the extended family of every newborn.

## Conclusion

Neonatal nurses are pillars of the NICU. Their knowledge and clinical skills help in providing the best practices to prevent ROP in preterm babies. Nurses ensure that services for preterm and low birth weight (LBW) babies include timely eye screening and organisation of follow up services.
